# Multi-Objective artificial bee colony optimized hybrid deep belief network and XGBoost algorithm for heart disease prediction

**DOI:** 10.3389/fdgth.2023.1279644

**Published:** 2023-11-16

**Authors:** Kanak Kalita, Narayanan Ganesh, Sambandam Jayalakshmi, Jasgurpreet Singh Chohan, Saurav Mallik, Hong Qin

**Affiliations:** ^1^ Department of Mechanical Engineering, Vel Tech Rangarajan Dr. Sagunthala R & D Institute of Science and Technology, Chennai, India; ^2^ School of Computer Science and Engineering, Vellore Institute of Technology, Chennai, India; ^3^ Department of Master of Computer Applications, MEASI Institute of Information Technology, Chennai, India; ^4^ Department of Mechanical Engineering and University Centre for Research & Development, Chandigarh University, Mohali, India; ^5^ Department of Environmental Health, Harvard T H Chan School of Public Health, Boston, MA, United States; ^6^ Department of Computer Science and Engineering, University of Tennessee at Chattanooga, Chattanooga, TN, United States

**Keywords:** heart disease, classification, deep belief network, XGBoost, feature selection, optimization

## Abstract

The global rise in heart disease necessitates precise prediction tools to assess individual risk levels. This paper introduces a novel Multi-Objective Artificial Bee Colony Optimized Hybrid Deep Belief Network and XGBoost (HDBN-XG) algorithm, enhancing coronary heart disease prediction accuracy. Key physiological data, including Electrocardiogram (ECG) readings and blood volume measurements, are analyzed. The HDBN-XG algorithm assesses data quality, normalizes using z-score values, extracts features via the Computational Rough Set method, and constructs feature subsets using the Multi-Objective Artificial Bee Colony approach. Our findings indicate that the HDBN-XG algorithm achieves an accuracy of 99%, precision of 95%, specificity of 98%, sensitivity of 97%, and F1-measure of 96%, outperforming existing classifiers. This paper contributes to predictive analytics by offering a data-driven approach to healthcare, providing insights to mitigate the global impact of coronary heart disease.

## 1. Introduction

Heart disease remains a leading health concern worldwide, particularly among adults and the elderly. As a condition that affects blood vessel function, it can lead to severe complications such as coronary artery infections. The World Health Organization (WHO) reports that heart diseases are the primary cause of death globally, accounting for approximately 30% of all fatalities (1). Given this alarming statistic, early prediction becomes paramount to effectively treat cardiac patients before the onset of heart attacks and strokes (2).

Predicting heart disease, however, is a complex task due to the myriad of contributing risk factors, including irregular pulse rate, high cholesterol, high blood pressure, diabetes, and several other conditions (3). Proper cardiac disease forecasting and timely warnings can significantly reduce the mortality rate. The creation of tools for predicting the risk of heart attacks relies on identifying and analyzing these risk variables, which can inform individuals about their potential vulnerabilities (4).

The realm of heart disease prediction has witnessed significant advancements, with researchers employing a myriad of techniques to enhance prediction accuracy. A common thread among these studies is the utilization of machine learning and optimization algorithms to achieve remarkable results. Several neural network and data mining techniques have been explored to enhance heart disease predictions. For instance, deep neural networks with dropout mechanisms have been employed to prevent overfitting, showing promise in improving prediction accuracy. However, the vast variety of instances in medical data and the broad spectrum of diseases and associated symptoms make comprehensive data analysis challenging.

Several recent studies have contributed amply to this area. MahaLakshmi and Rout (5) proposed an ensemble-based IPSO model, achieving an impressive 98.41% accuracy on the UCI Cleveland dataset. Similarly, Mohapatra et al. (6) utilized stacking classifiers for their predictive model, achieving 92% accuracy. Chandrasekhar and Peddakrishna (7) further enhanced prediction using a soft voting ensemble classifier, marking an accuracy of 95% on the IEEE Dataport dataset. Optimization techniques have also been at the forefront of these advancements. Takcı et al. (8) optimized the KNN algorithm using genetic algorithms, achieving 90.11% accuracy on the Cleveland dataset. Fajri et al. (9) explored the bee swarm optimization algorithm combined with Q-learning for feature selection, outperforming many existing methods.

Few researchers have also employed deep learning approaches to make accurate prediction relating to heart disease. Dhaka and Nagpal (10) presented a model using deep BiLSTM combined with Whale-on-Marine optimization, achieving 97.53% accuracy across multiple datasets. Bhavekar and Goswami (11) introduced the travel-hunt-DCNN classifier, marking 96.665% accuracy on a specific dataset. Jayasudha et al. (12) further developed a hybrid optimization deep learning-based ensemble classification, achieving a commendable 95.36% sensitivity.

Still fewer have used hybrid and specialized approaches for heart disease prediction. Saranya and Pravin (13) combined the Random Forest classifier with hyperparameter tuning, achieving up to 96.53% accuracy. Asif et al. (14) utilized the extra tree classifier in their machine learning model, achieving 98.15% accuracy. Krishnan et al. (15) proposed a model using transfer learning and hybrid optimization, emphasizing both reduced training time and improved accuracy. Yaqoob et al. (16) presented a unique hybrid framework addressing both privacy concerns and communication costs, improving prediction accuracy by 1.5%. Rajkumar et al. (17) ventured into IoT-based heart disease prediction using deep learning, marking 98.01% accuracy. Kiran et al. (18) specifically explored the effectiveness of machine learning classifiers for prediction CVD, proposing the GBDT-BSHO approach and achieving 97.89% accuracy.

In this research, we introduce a novel classifier, the Hybrid Deep Belief Network and XGBoost (HDBN-XG) technique, aiming to offer a more precise prognosis of heart disease. This method stands out by leveraging advanced machine learning algorithms to analyze and predict heart disease risks more effectively than traditional methods.

The remainder of this paper is structured as follows: Part II reviews relevant works in the domain of heart disease prediction. Part III delves into the proposed HDBN-XG technique. Part IV presents a comprehensive performance analysis, and Part V concludes the study with key findings and future directions.

## 2. Methods

**Figure 1:**
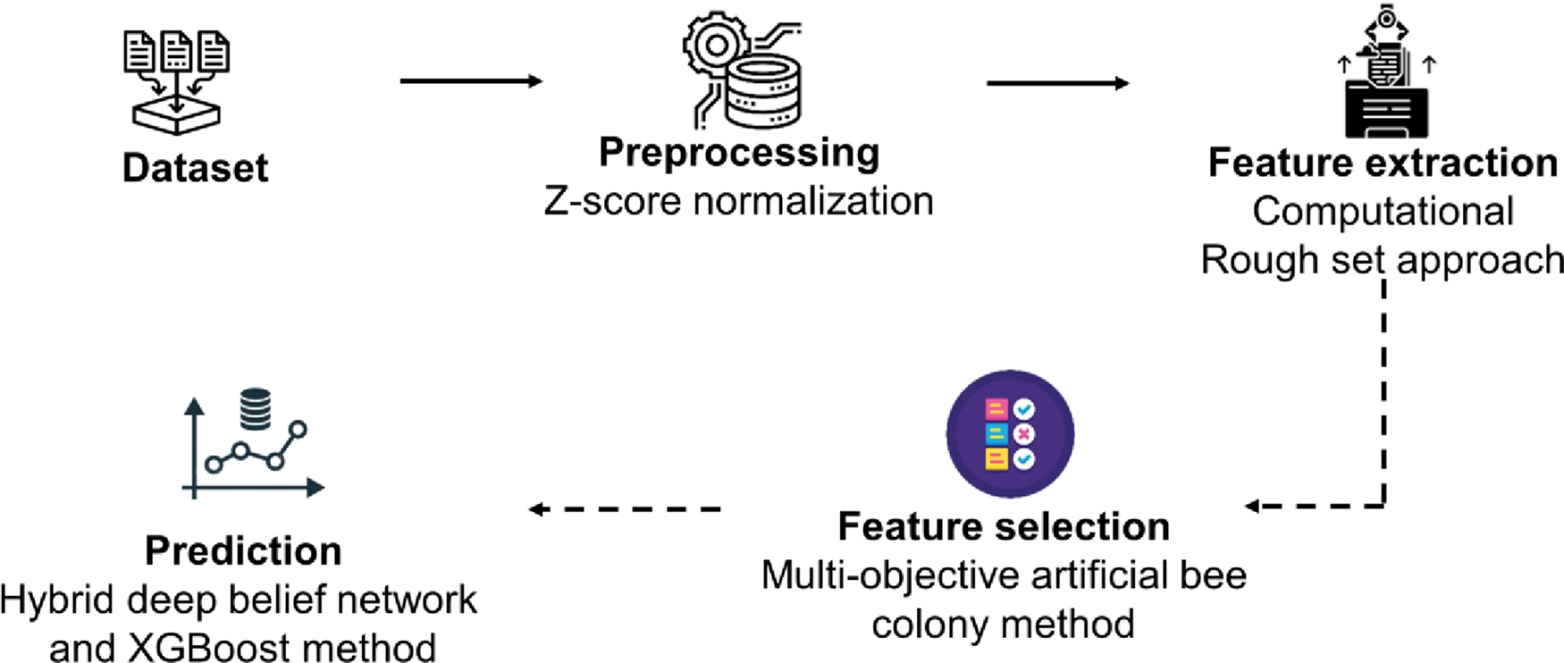
Flowchart of the proposed methodology.

The methodology of the proposed technique is explained in this section. The process flow diagram for the proposed method illustrates the review of wearable devices, gateway, cloud platforms, medical history, data collection analysis for heart disease prediction, feature extraction using the computational rough set method, preprocessing using z-score normalization, feature selection using the multi-objective artificial bee colony method, hybrid deep belief network, and XGBoost method, among other processes. Figure 1 shows a schematic illustration of the recommended approach.

### 2.1. Dataset collection

This study used data from the smaller heart diseases in South Africa data collections spe-cifically focusing on Coronary Heart Disease (CHD). The dataset comprises 462 occurrences (observations), 10 attributes (nine of which are independent variables) and 1 variable, as shown in Table 1. (CHD, the labeled class). KEEL is the recollective sample of males from Western Cape of South Africa, a region with a high prevalence of cardiovascular disease. Positive (1) and negative (0) results are predicted for the designated class CHD, respectively (19).

**Table 1:** Attributes description of the KEEL dataset.

Attribute	Domain	Data type	Missing value?
CHD (class)	(0,1)	Binary	No
Obesity	[14.7,46.58]	Decimal	No
Age	[15,64]	Decimal	No
Types	[13,78]	Decimal	No
Adiposity	[6.74,42.49]	Decimal	No
Ldl	[0.98,15.33]	Decimal	No
Tobacco	[0.0,31.2]	Decimal	No
Sbp	(101,218)	Decimal	No
Alcohol	[0.0,147.19]	Decimal	No
Famhist	{Present, Absent}	Text	No

The selected variables are based on extensive literature review and their proven association with coronary heart disease. For instance, the “Type-A behavior” variable has been linked to heart diseases in various studies due to its association with stress and aggressive behavior (20, 21). Following up on each high-risk patient, the following traits were noted: Some of the variables taken into account include systolic blood pressure (sbp), lifetime tobacco use measured in kilograms (tobacco), low-density lipoprotein cholesterols (ldl), bad cholesterol, adiposity, family history for heart diseases (famhist), type-A personality (typea), obesity, current alcohols consumptions (alcohol), and age at onset (age).

We define a few terms below in order to provide a clear understanding.

• Sbp: When the heart is beating, the blood pressure is that matters.
• Adiposity: It is calculated as a body fat percentage.
• Type-A behavior: It's a quality of an aggressive, impatient, and competitive person.
• Obesity: By dividing the person's weight by their height squared, the Body Mass Indexes (BMI), that measures it, is obtained.

The first five examples of the datasets under investigation are shown in Table 2.

**Table 2:** CHD dataset sample instances of the KEEL dataset.

sbp	Tobacco	Ldl	Adiposity	Famhist	Types	Obesity	Alcohol	Age	CHD
160	12	5.73	23.11	Present	49	25.3	97.2	52	YES
144	0.01	4.41	28.61	Absent	55	28.87	2.06	63	YES
118	0.08	3.48	32.28	Present	52	29.14	3.81	46	NO
170	7.5	6.41	38.03	Present	51	31.99	24.26	58	YES
134	13.6	3.5	27.78	Present	60	25.99	57.34	49	YES

### 2.2. Preprocessing using Z-score normalization

The produced data must be normalized using the Z-score Normalization technique before employing the computational rough set approach. The requested range may be extracted from the dataset using this approach, which is based on the data's mean and standard deviation. It was discovered that using this technique might improve the model's accuracy. Eq. 1 displays the formula of Z-score normalization (22).

(1) $$X_{i}^{\prime }=\frac{x_{i-\mu }}{\sigma }$$

Where $X_{i}^{\prime }$ is the normalized data, $x_{i}$ = Original data, μ = Average of data, σ_ _= Standard deviation of data.

### 2.3. Feature extraction of computational rough set approach

The relevant qualities are evaluated using the notion of reducts or core given by rough set theory. This indiscernibility connection makes it simpler to find duplicate values or redundant properties in a set. The numerous set approximation subset of characteristics that appear in minimum are known as reductions. A core is the set of all conditional qualities of set approximations which exist as a set, and is defined as intersection of all reductions to a set or a system taken into account (23).

For instance, the diagram appears as follows if A is a set of characteristics and B is a subset of e. According to the Eq (2).

(2) $$A=(U,r,d)\&P_{R}((d)=P_{C}(d)$$

If core × specifies all conditional attributes and core Y specifies the whole set of reducts of attribute Z. Using dynamically produced decision tables is one way to compute these reducts or conditional characteristics. In these choice tables, the qualities are given in two different ways: significant and often. The group of qualities that tend to be shared by original sets in decision table is given precedence when they are repeated often and are given the status for majority or substantial. The rough set theory concepts core and reduce provide the foundation for the proposed rough computational intelligence-based attribute selection method (23).

The elimination of pointless data from a decision table or information table without having an impact on the remaining data in the table is referred to as the removal of significant characteristics. As a consequence, the elimination of superfluous characteristics is generalized using the value of attributes. Attributes must first be evaluated in order to establish their value. The process of gaining important attributes in a decision table may be finished by deleting attributes from the attribute collection. Let the attribute be in a set for a set that is regarded to be β(r,e). And when attribute an is taken out of the set β(r,e), it may be specified as Eq. (3),

(3) β((r⇔a,e))

The relevance of characteristics may then be determined using the aforementioned requirements and procedures by normalizing the fundamental difference between the coefficient and the set produced after the attribute has been removed. i.e; β(r,e) and β((r⇔a,e)). The Eq. (4) is described below.

(4) α(r,e)(a)=(β(r⟨=⟩a,e))/(β(r,e))

Therefore, in this case, we refer to the coefficient A as the error of classification. If the attribute is not included in the set under consideration, a misclassification will result. As a result, the importance of an attribute set may be expanded by the remaining characteristics in the set, and expressed as Eq. (5).

(5) α(r,e)(x)=(β(r,e))⟨=⟩(β(r⟨=⟩x,e))/(β(r,e))

The coefficient resulting from the extension of a attribute significance is indicated here as α(x). Additionally,  × is regarded as a part of r, i.e the collection of qualities in r are reduced to x. After eliminating the attribute, this may be written as, where every subset × and r is regarded as the reduct of r. The Eq (6) is given below,

(6) α(r,e)(x)=(β(r,e))⟨=⟩β(x,e)/(β(r,e))

As a result, the definition of α(r,e) is the reduct approximation or inaccuracy of reduct approximation that illustrates the relevance of × qualities in relation to r. The least approximation error improves accuracy in a series through a classification approach. The most significant traits that cause heart disorders in the health sector are discovered using the suggested Rough Computational Intelligence based Attribute Selection approach on heart disease data sets.

### 2.4. Feature selection of multi-objective artificial bee colony method

A bionic intelligence system called the Multiobjective Artificial Bee Colony algorithm (MABC) models how honeybees gather honey. The worker bee, observer bee, and scout bee are three of the bee species that are included in the algorithm's fundamental models of sources and bees. The model simultaneously identifies two behaviours: enlisting bees to defend food sources and leaving food sources. The three types of bees each carry out distinct tasks, but they also cooperate to swiftly and correctly find and gather food sources. The following Eq (7) represents a general multi-objective optimization problem.

$$minJ=[J_{1}(x),J_{2}(x),\ldots .J_{N}(x)]$$

(7) $$s.t:\;X_{min}\leq x\leq X_{max},i=1,\ldots .,m$$

Where $X_{min}$ and $X_{max}$ represent the lower and upper limits, respectively, and × is an m-dimensional choice variable. The vector of the objective function is J. A multi-objective optimization issue exists when N≥2. The solutions may be classified as feasible and infeasible depending on whether a constraint is met or not, making it easier to solve the constraint issue.

The multiobjective artificial bee colony method central tenet is the importance of transformation, work division, and collaboration among various bee species. There are three approaches to evolve solutions in the multiobjective artificial bee colony (MABC) method.

#### 2.4.1. Solutions evolve in employed Bee

The following formula (8) illustrates how the original solution is generated via the use of employed bees.

(8) $$x_{i,d}^{new}=x_{i,d}+\phi_{i,d}.(x_{i,d}-x_{k,d})$$

Where $\phi_{i,d}$ denotes the rate of solution change and $x_{k,d}$ is adjacent ’s food supply's d-dimensional variable $x_{i,d}$.

Local evolution and this form of evolution methodology are related. To determine whether or not to replace the previous solution after acquiring a new one, it is important to assess the objective function.

#### 2.4.2. Onlooker Bee solutions

At this point, the hired bee is picked by the spectator bee using a random number generator. Accordingly, the more nectar the employment bee's related food source has, the better the quality of a viable solution is, and the more likely it is to be chosen. In order to undertake local searches and evolutions around a food supply and create new, higher-quality individuals, the observer bees employ the following formula (9).

(9) $$x_{i,d}^{new}=x_{i,d}+\phi_{i,d}.(x_{i,d}-x_{q,d})$$

Where $x_{q}$ stands for an alternative food supply to $x_{k}$.

#### 2.4.3. Solutions evolve in scout Bee

Updates to the solutions are found using the scout bee. After multiple evolutions, if a food supply has not been changed, it stops using it when it reaches a certain threshold, called Limit, and create sources at random to prevent prematurely entering local optimization. The Pareto dominance technique is often employed for ranking in multi-objective optimization situations. If $J(x_{1})$ objective's function is better to or equal to the analogous component in $J(x_{2})$ and there is at least one objective function that is strictly superior to $J(x_{2})$, then one viable solution $x_{1}$ dominates another feasible solution in a problem solution set. Two viable solutions are said to be non-dominant if they do not conflict with one another.

**Figure 2:**
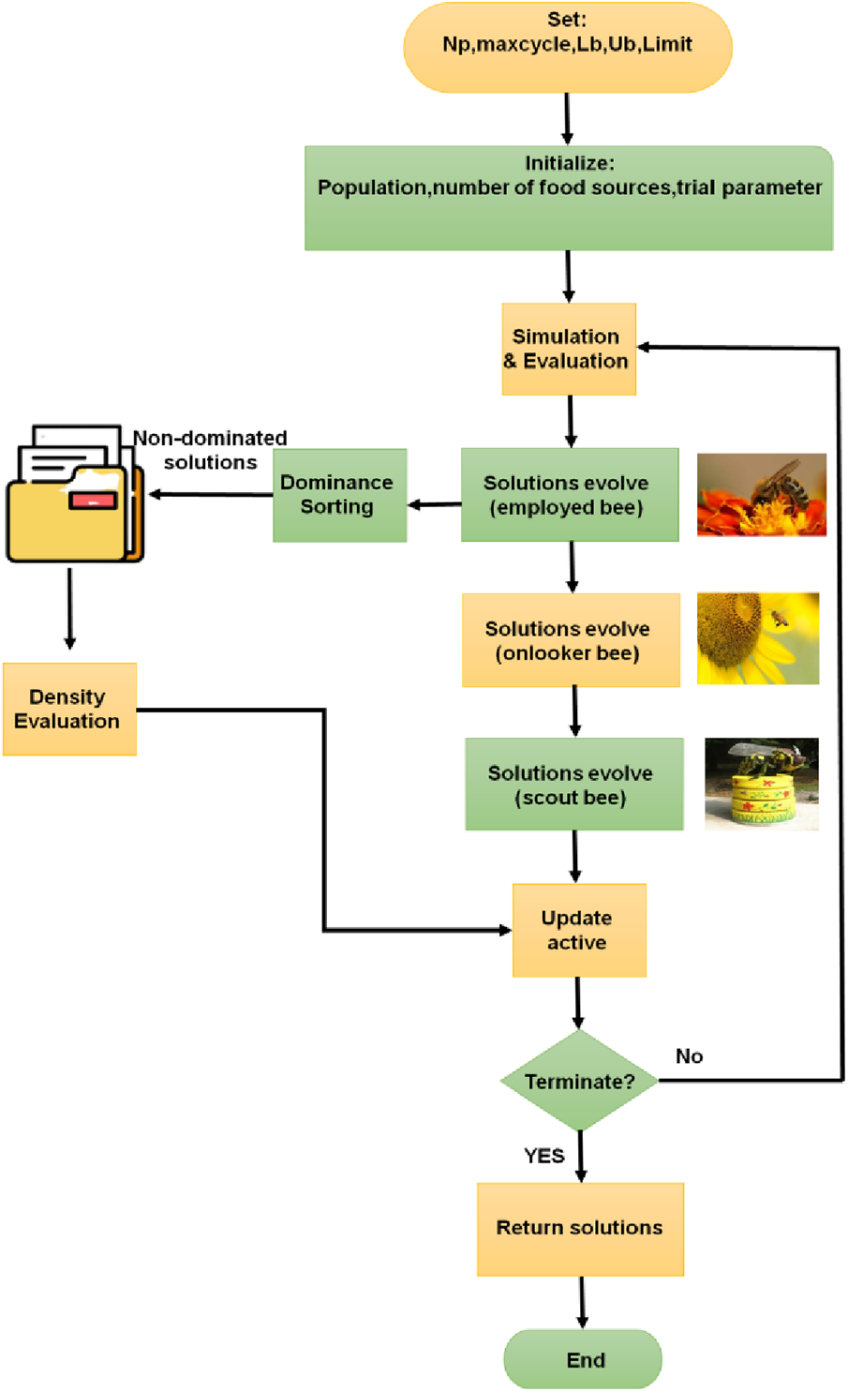
Representation of the MABC algorithm.

First, a population size, maximum numbers of cycles, and upper and lower bounds of the optimization variable referred to as Np, max cycle, Ub and Lb, need to be specified for the MOABC method. The first solution is then created at random in the initial solution space. The aforementioned evolution strategy results in iterative optimization and Pareto dominated sorting. Density evaluation spreads non-dominated solutions uniformly over the Pareto front to avoid method settling. Figure 2 depicts the method for the artificial bee colony.

### 2.5. Hybrid deep belief network and XGBoost method

Due to its semi-supervised learning techniques, the hybrid deep belief network (HDBN) is a machine learning algorithm that has gained popularity. The learning method for the DBN consists of two stages: unsupervised learning and supervised learning. Using stacked Restricted Boltzmann Machines that have undergone an unsupervised pre-training, the first step assesses the weights and biases between visible and hidden layers (RBM). Between two adjacent visible-hidden layers or hidden-hidden layers, RBMs are layered. RBMs only link neighboring nodes since they are energy-based functions. The likelihood of greedy layer-wise approach is used to assess weights and biases between hidden and visible layers. In the second step, pre-training is followed by supervised parameter improvement using weighted neurons and biases.

The hybrid deep belief network (HDBN) is a customized model with a large number of hidden DL layers. In comparison to lower levels, the higher layers of the DBN may include more specific and descriptive characteristics to pinpoint the prediction of predictive systems. The DBN offers more significant benefits than the standard neural networks, including the capacity to use the connections between the features in more complex processes and obtaining excellent performance with less training sets. Weights and biases are adjusted via fine-tuning during the supervised learning phase, which uses the gradient descent or ascent algorithms to increase the accuracy and sensitivity of models. The DBN is a probabilistic joint distribution of the *l* hidden layers and the input vector *x* as follows Eq (10).

(10) $$P(x,h^{1},\ldots ,h^{l})=\left(\prod_{k=0}^{t-2}P(h^{k}|h^{k+1})\right)P(h^{k-1},h^{l})$$

Where $h^{0}$ is the input vector, and $P(h^{k-1},h^{l})$ is the probability of the conditional distribution among the neighbouring layers.

As described below the Eq (11), state $\left(h^{k-1},h^{k}\right)$ energy function is

(11) $$E(h^{k-1},h^{k};\theta )=-\sum_{s=1}^{D_{k-1}}\sum_{t=1}^{D_{k}}W_{st}^{k}h_{s}^{k-1}h_{t}^{k}-\sum_{s=1}^{D_{k-1}}b_{s}h_{s}^{k-1}-\sum_{t=1}^{D_{k}}c_{t}h_{t}$$

Where $\theta =(w_{st},b,c)$ that are a DBN's parameters; the weight between the $s^{th}$ neuron in layer $h^{k-1}$ and the $t^{th}$ neuron in layer $h^{k}$ is called $W_{st}^{k}$. $D_{k}$ represents the quantity of neurons in a $k^{th}$ layer. Eq (12) describes the probability distribution of the energy function.

(12) $$P(h^{k-1};\theta )=\frac{\sum_{h}k\exp(-E(h^{k-1},h^{k};\theta ))}{\sum_{h}k-1\;\sum_{h}k\exp(-E(h^{k-1},h^{k};\theta )}$$

The estimated weights are adjusted using supervised learning based on gradient descent after layer-wise unsupervised learning. *w* parameters are updated throughout this fine-tuning procedure to improve classification results and discriminative power.

One type of neural network called a DBN comprises of several Restricted Boltzmann Machines (RBMs), each of which includes an input visible layer IV and an output hidden layer OH: Although there is no link between the inner levels, these layers are completely interconnected. Here, RBM uses an energy function Eng(v,h) that is defined in Eq. (13) to learn the probability distribution from the input visible layer to the output hidden layer.

(13) $$Eng(v,h)=-\sum_{P=1}^{q}a_{P}iv_{p}-\sum_{s=1}^{m}b_{s}oh_{s}-\sum_{P=1}^{q}-\sum_{s=1}^{m}oh_{s}W_{\;ps}iv_{p}$$

Based on the hidden unit $IV(iv_{1},\ldots .,iv_{m})$ and the visible unit $OH(oh_{1},\ldots ,oh_{q})$, energy is calculated, and the connection weight between each layer is reported as $W_{\;ps}$. Matching nodes' bias terms are denoted by the symbols $a_{P}$ and $b_{P}$, respectively. The partition function Y from Eqs (14) and (15) defines the probability distributions p(v,h) over hidden unit $IV(iv_{1},\ldots .,iv_{m})$ and visible unit $OH(oh_{1},\ldots ,oh_{q})$.

(14) $$\rho (v,h)=\frac{e^{-Eng(v,h)}}{Y}$$

(15) $$Y=\sum_{iv}\sum_{oh}e^{-Eng(v,h)}$$

The formulation of the individual activation probability, $p(v_{p}=1|h)$ is provided in Eqs (16) and (17).

(16) $$(v_{p}=1|h)=AF\left(b_{s}+\sum_{\;p=1}^{m}W_{\;ps}oh_{s}\right)$$

(17) $$p(v_{s}=1|h)=AF\left(b_{s}+\sum_{\;p=1}^{m}W_{\;ps}oh_{s}\right)$$

The activation function or logistic sigmoid function is referred to as AF in this context.

A HDBN is constructed using a greedy layer-wise method from a stack of RBMs. Here, it is encouraged to use unlabeled data effectively based on the theory of learning. Pretraining and fine tuning in training are the two main aspects of HDBN. RBMs are trained and achieve criteria like weight and bias terms during the pre-training stage. Second, a back-propagation mechanism is used to fine-tune the parameters during the fine-tuning phase. Additionally, RBMs are capable of identifying and extracting characteristics based on many layers of RBMs, where every layer uses the hidden neurons from the layer underneath it as an input. In the HDBN, RBM layers are utilized for feature detection while a multilayer perceptron is used for prediction.

The ensemble tree approaches XGBoost (Extreme Gradient Boosting) and Gradient Boosting (GB) both employ the gradient descent architecture to strengthen weak learners. However, the fundamental GB architecture is strengthened by XGBoost thanks to system optimization and algorithmic upgrades. A software that is a part of the Distributed Machine Learning Community is called XGBoost (DMLC). Stage-wise additive modelling is what GB does. An inadequate classifier is first fitted to the data. Without altering the first classifier, it is fitted with a second weak classifier to enhance the performance of the existing model. Every new classifier must take into account the areas in which the older ones struggled. According to the following Eq. (18),

(18) $$D=X;y,|D|=n,x\in R^{m},y\in R$$

The dataset's samples, features, and target variable are indicated by the notation n samples, m features, and. Our heart disease dataset has n=303 observations, m=13 characteristics, and n variables. According to Eq. (19), the prediction outcome for dataset D in GB is represented by the total of the k trees predicted scores, which is determined using the K additive function.

(19) $${\hat{y}}_{I}=\sum_{k=1}^{k}f_{k}(x_{i}),f_{k}\in F$$

The loss function $L_{k}$, which is described in Eq. (20), is minimised by GB.

(20) $$L_{k}=\sum_{l=1}^{n}L({\hat{y}}_{i},y_{i})$$

Since GB and XGBoost are tree-based algorithms, many tree-related hyper-parameters are used to reduce overfitting and improve model performance. The learning rate influences the model's tree weighting and adaptation to training data. Add the regularization term and loss function to get XGBoost's objective function. Loss function controls the model's forecasting performance, whereas regularization controls its simplicity. Eq. (21) serves as a definition of the XGBoost's goal function.

(21) $$Obj=\sum_{l=1}^{n}L({\hat{y}}_{i},y_{i})+\sum_{l=1}^{k}R(\;f_{i})$$

Gradient descent is used by XGBoost to optimise the objective function (24). Our model is additive; therefore, a tree is added if the forecast matches the total of the previous and new tree's results. Column sub sampling is used in XGBoost to reduce over fitting alongside GB. Using column sub sampling reduces over fitting.

## 3. Results and discussion

In this section, we discuss the proposed framework and its overall behavior. For our experiments, the dataset was divided into a training set and a testing set. 80% of the data (369 observations) was used for training the model, and the remaining 20% (93 observations) was used for testing its performance. This ensured that our model was evaluated on unseen data, providing a realistic assessment of its predictive capabilities.

### 3.1. Selected features

In our study, the Multi-Objective Artificial Bee Colony method was employed to select the most relevant features from the dataset. The method evaluates the importance of each feature based on its contribution to the prediction accuracy and reduces the dimensionality of the dataset by retaining only those features that significantly influence the outcome. After applying the feature selection method, we retained 8 out of the initial 9 features. The retained features were sbp, tobacco, ldl, adiposity, famhist, types, alcohol and age. These features were then used in the subsequent modeling process. The feature “obesity” was dropped from the dataset.

### 3.2. Accuracy

The capacity of a test to accurately distinguish between patients and healthy instances is a measure of its accuracy. Calculating the percentage of true positive and true negative results in all analysed instances is necessary to measure a test's accuracy. The accuracy Eq. (22) is described given below

(22) Accuracy=(TP+TN)/(TP+TN+FP+FN)

**Figure 3:**
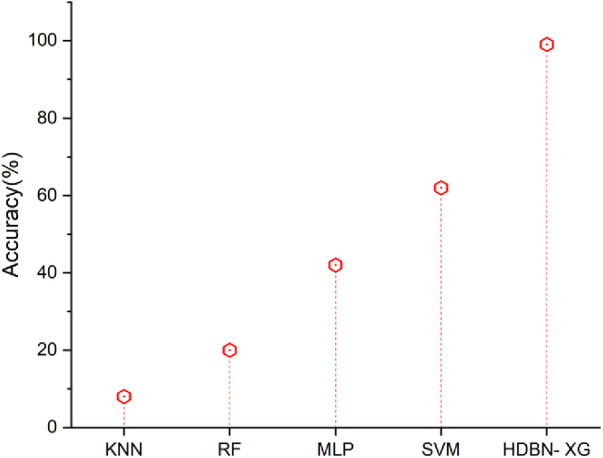
Accuracy of the proposed HBDN-XG with other popular ML techniques (SVM (25), KNN (26), MLP (27), RF (28)).

Figure 3 represents that the accuracy results of proposed and existing methodology. In terms of accuracy the proposed method of hybrid deep belief network and XGBoost method have 99% and the existing methods of k-nearest neighbor have 8%, random forest have 20%, multilayer perceptron have 42%, support vector machine have 62%, so when compared to existing methods the proposed technique perform high in terms of accuracy.

### 3.3. Precision

In a two-class imbalanced classification problem, precision is calculated as the number of true positives divided by the total of true positives and false positives. The precision Eq. (23) is described given below

(23) $$Precision\;=\;\frac{TP}{TP+FP}$$

**Figure 4:**
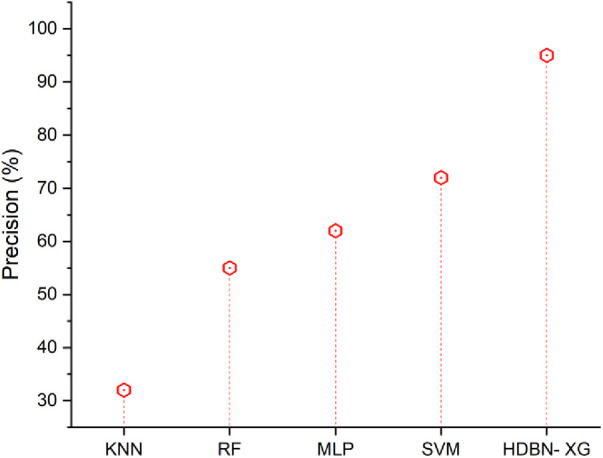
Precision of the proposed HBDN-XG with other popular ML techniques (SVM (25), KNN (26), MLP (27), RF (28)).

Figure 4 displays the precision outcomes using both the proposed and existing approaches. In terms of precision the proposed method of hybrid deep belief network and XGBoost have 95% and the existing methods of k-nearest neighbor have 32%, random forest have 55%, multilayer perceptron have 62%, support vector machine have 72%, so when compared to existing methods the proposed technique perform high in terms of precision.

### 3.4. Specificity

The ability of a test to recognize healthy samples serves as a gauge of its specificity. In order to calculate an estimate, we should determine the actual negative proportion under healthy conditions. The following Eq. (24) can be expressed.

(24) $$Specificty\;=\frac{TN}{TN+FP}$$

**Figure 5:**
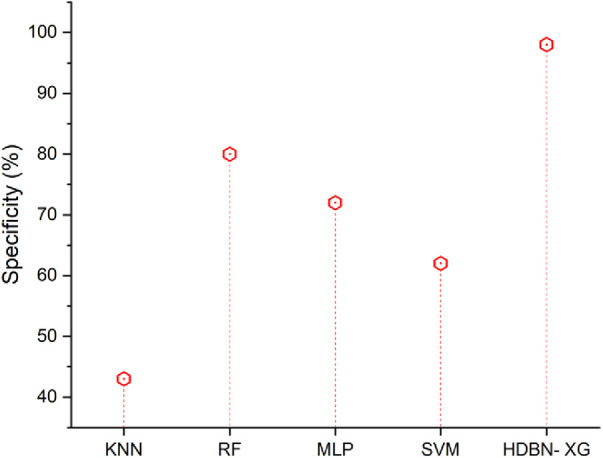
Specificity of the proposed HBDN-XG with other popular ML techniques (SVM [25], KNN [26], MLP [27], RF [28]).

Figure 5 shows that, when compared to a proposed technique, suggested methods including SVM, MLP, RF, and KNN have low specificity values. In terms of specificity the proposed method of hybrid deep belief network and XGBoost have 98% and the existing methods of k-nearest neighbor have 43%, random forest have 80%, multilayer perceptron have 72%, support vector machine have 62%, so when compared to proposed method the existing techniques perform low in terms of specificity.

### 3.5. Sensitivity

Sensitivity in medicine is the proportion of those who test positive for an illness who really have that sickness. Those who do not have the illness will basically be ruled out by a very sensitive test. Frequently, screening tests that are very sensitive are employed. The Eq. (25) is calculated follows as,

(25) $$Sensitivity=\;\frac{TP}{TP+FN}$$

**Figure 6:**
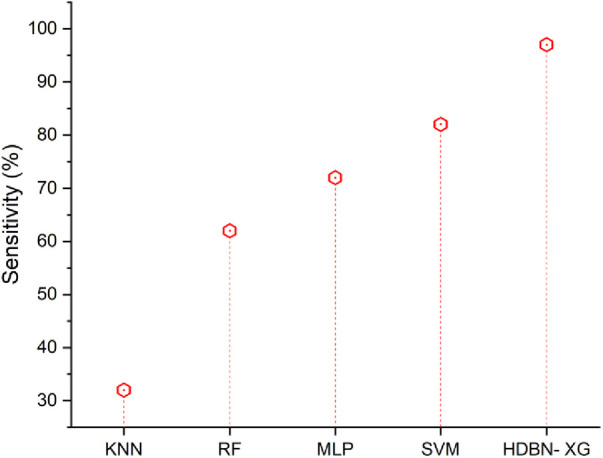
Sensitivity of the proposed HBDN-XG with other popular ML techniques (SVM (25), KNN (26), MLP (27), RF (28)).

As shown in Figure 6, the suggested approach of HDBN-XG has a high sensitivity than the existing methods. In terms of sensitivity the proposed method of hybrid deep belief network and XGBoost have 97% and the existing methods of k-nearest neighbor have 32%, random forest have 62%, multilayer perceptron have 72%, support vector machine have 82%, so when compared to proposed method the existing techniques perform low in terms of sensitivity.

### 3.6. F-measure

The F-measure represents a happy medium between recall and precision. In terms of measuring success, it is a statistic. A person's F-measure represents the mean of their accuracy and sensitivity scores. The Eq. (26) is described below

(26) $$F-measure=\frac{TP}{TP+\frac{1}{2}(FP+FN)}\;$$

**Figure 7:**
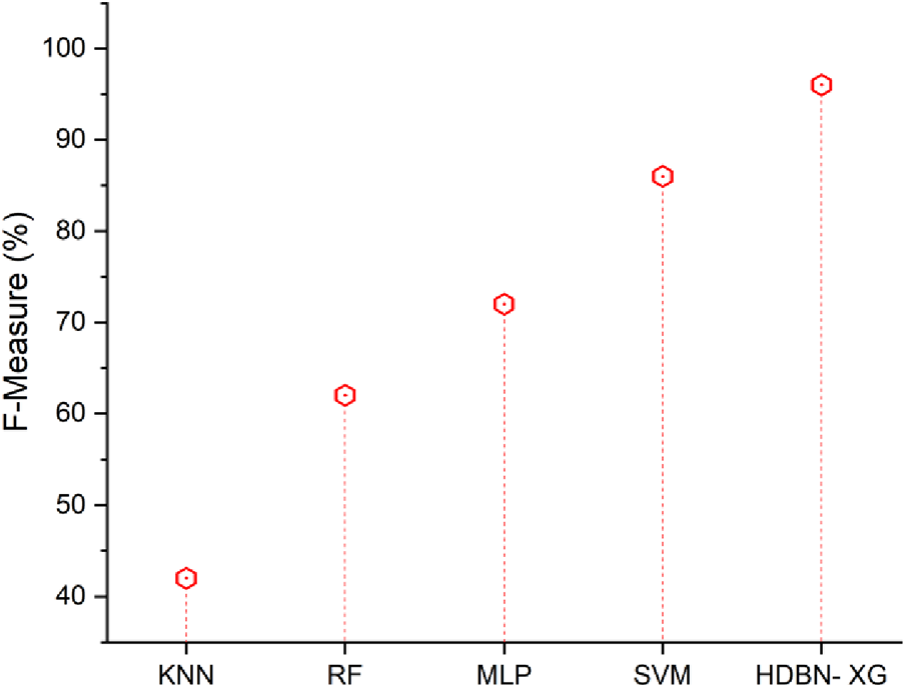
F-measure of the proposed HBDN-XG with other popular ML techniques (SVM (25), KNN (26), MLP (27), RF (28)).

Figure 7 represents the F-measure results of the proposed and existing methodology. From Figure 7 the proposed approach has a high f-measure than the existing methods. In terms of F-measure the proposed method of hybrid deep belief network and XGBoost have 96% and the existing methods of k-nearest neighbor have 42%, random forest have 62%, multilayer perceptron have 72%, support vector machine have 86%, so when compared to existing methods the proposed technique perform high in terms of F-measure. When compared to existing methods, the analysis and comparison for all parameters of a proposed method has a high percentage.

### 3.7. Discussion

As seen above the proposed HBDN-XG is compared with SVM (25), KNN (26), MLP (27) and RF (28). KNN is a supervised learning classifier that employs proximity to produce classifications or predictions about the grouping of a single data point. It is simple to use and comprehend; it slows down when more data is used. Its main flaws are computational inefficiency and difficulty choosing K. As an ensemble learning technique for classification, regression, and other problems, random forests build a large number of decision trees during the training phase. The biggest drawback of random forest is that it might be too sluggish and inefficient for real-time forecasts when there are a lot of trees. These algorithms are often quick to train but take a long time to make predictions after training. A feedforward neural network class that is completely linked is called an MLP. When used ambiguously, the word MLP might apply to any feedforward neural network or specifically to networks made up of several layers of perceptrons. The multilayer perceptron's drawback is that it is unknown how much each independent variable influences the dependent variable. Calculations are challenging and time-consuming. SVM is a well-known Supervised Learning technique that may be used to both classification and regression tasks. In Machine Learning, however, its primary use is in the realm of Classification. When there is a lot of overlap between the target classes in the data set, SVM struggles to perform effectively.

On the other hand, deep belief networks have the benefit of effectively using hidden layers (higher performance gain by adding layers compared to Multilayer perceptron). DBN provides a unique level of classification resilience (size, position, color, view angle—rotation). Gradient Boosting comes with a simple to understand and comprehend method, making most of its forecasts straightforward to manage. XGBoost excels on structured datasets with somewhat few characteristics and on small datasets that include subgroups. So, to overcome the existing issues we used the hybrid deep belief network and XGBoost method in this work.

## 4. Conclusion

In the pursuit of advancing heart disease prediction, our research introduced the Hybrid Deep Belief Network and XGBoost (HDBN-XG) technique. This method was developed to provide a more precise prognosis of heart disease, a critical factor in effective treatment before severe cardiac events. Based on the study, the following main conclusions can be drawn—

• The HDBN-XG prediction system achieved an impressive accuracy of 99%, precision of 95%, specificity of 98%, sensitivity of 97% and the F1-measure stood at 96%.
• The proposed HDBN-XG method consistently outperformed current classifiers like SVM, MLP, RF and KNN in all evaluated parameters, indicating its potential as a leading tool in heart disease prediction.

In light of these findings, the HDBN-XG technique holds significant promise for the healthcare sector, offering a robust tool for early and accurate heart disease prediction. The implications of such a tool are vast, from timely interventions to better patient management. As we look to the future, we aim to further refine and enhance the performance of this predictive classifier. Exploring different feature selection methods and optimization techniques will be pivotal in this journey. Moreover, the potential integration of our approach with healthcare systems could revolutionize patient care, ensuring timely and effective treatments. Collaborations with healthcare practitioners and policymakers will be essential to maximize the impact of our research, ultimately aiming to mitigate the global challenge posed by heart disease.

## Data Availability

The raw data supporting the conclusions of this article will be made available by the authors, without undue reservation.
